# Anaesthetic challenges in a nonagenarian with Parkinson’s disease undergoing hip fracture surgery: a case report

**DOI:** 10.1186/s12871-026-04126-x

**Published:** 2026-07-23

**Authors:** Pushkar Bishwokarma, Rabi Paudel, Shova Dangol

**Affiliations:** Department of Anaesthesiology, Armed Police Force Hospital, Kathmandu, Nepal

**Keywords:** Geriatric anaesthesia, Hip fracture, Hypertension, Hypothyroidism, Intrathecal dexmedetomidine, Language barrier, Multimorbidity, Nonagenarian, Parkinson’s disease, Postoperative delirium

## Abstract

**Background:**

Hip fractures in nonagenarian patients with multiple comorbidities present substantial anaesthetic challenges. Parkinson’s disease (PD) adds further complexity through motor symptoms, autonomic dysfunction, and the imperative for uninterrupted dopaminergic therapy.

**Case presentation:**

We report the perioperative anaesthetic management of a 93-year-old male with hypertension, Parkinson’s disease, hypothyroidism, and dyslipidaemia who underwent cemented left hemiarthroplasty for a transcervical neck of femur fracture. The case was additionally complicated by a significant language barrier, as the patient was exclusively fluent in Newari. Spinal anaesthesia was administered at L3–L4 using 3 mL of 0.5% hyperbaric bupivacaine with 5 µg intrathecal dexmedetomidine, selected to prolong anaesthesia based on discussion with the orthopaedic team; the rationale and limitations of this dosing strategy are critically appraised in the Discussion. Intraoperatively, the patient developed three episodes of hypotension, likely related to sympathetic blockade and adjuvant effects, managed with incremental doses of mephentermine. An episode of intraoperative hypoglycaemia (blood glucose 55 mg/dL) was promptly corrected with 50% dextrose. Postoperatively, the patient developed transient hypoactive delirium due to temporary interruption of dopaminergic therapy, which resolved after resumption of enteral medications by postoperative day 2. On postoperative day 3, the patient developed aspiration pneumonia after oral feeding was initiated by family members despite a functioning nasogastric tube. The condition was managed with antibiotics and supportive care. The patient subsequently improved and was discharged on postoperative day 10.

**Conclusion:**

Spinal anaesthesia is feasible in nonagenarians with multiple comorbidities, including Parkinson’s disease. This case highlights the need for evidence-based dose optimisation and proactive complication prevention. It further emphasises the importance of low-dose bupivacaine–based spinal anaesthesia in very elderly patients, meticulous perioperative glycaemic monitoring, continuation of dopaminergic therapy, structured delirium prevention strategies, caregiver education, and strict adherence to dysphagia-safe feeding protocols in high-risk geriatric patients.

**Supplementary Information:**

The online version contains supplementary material available at https://doi.org/10.1186/s12871-026-04126-x.

## Introduction

Hip fractures affect over 1,600,000 individuals worldwide annually, with projections estimating 4.5 million cases by 2050, and nonagenarians carry a disproportionate mortality burden, with a one-year mortality rate of 38.1% and an overall perioperative complication rate of 44.1%, substantially exceeding the 21% one-year mortality reported for elderly hip fracture patients overall. [1] Anaesthetic management in nonagenarian patients is demanding: reduced physiological reserve, multiple comorbidities, and heightened susceptibility to cardiovascular instability, respiratory compromise, and postoperative delirium, all narrow the safety margin. [2].

Parkinson’s disease (PD) is associated with significant perioperative risk, including motor dysfunction, autonomic instability, and dopaminergic vulnerability. [3] Oropharyngeal dysphagia occurs in 40–80% of patients due to bradykinesia and rigidity of pharyngeal musculature, and aspiration pneumonia remains the leading cause of disease-related mortality. [4] A key clinical issue is that levodopa/carbidopa is only available orally; therefore, postoperative nausea and vomiting (PONV) can directly interrupt drug delivery and precipitate acute motor deterioration or rigid crisis. Opioids, particularly fentanyl, are well-recognised triggers of PONV and should be used cautiously in PD patients due to this risk of impaired medication absorption. [3] Gastrointestinal dysfunction is also common, with nausea, vomiting, and constipation occurring in up to 81% of patients, reflecting underlying autonomic and enteric nervous system involvement. Accordingly, dysphagia screening and timely dietary modification should be implemented at the preoperative stage rather than being initiated after the development of complications. [3].

Regional anaesthesia is generally preferred in high-risk elderly patients because it avoids airway manipulation, reduces systemic drug exposure, preserves gastrointestinal motility via sympathetic blockade, attenuates the surgical stress response, reduces opioid requirements, and lowers PONV incidence, all of which are particularly valuable in patients with Parkinson’s disease who depend on uninterrupted enteral dopaminergic therapy. [5] We report the anaesthetic management of a 93-year-old patient with PD, hypertension, hypothyroidism, dyslipidaemia, and a significant language barrier who underwent cemented hemiarthroplasty (HA) for a transcervical neck of femur fracture under spinal anaesthesia.

## Case presentation

### Preoperative assessment

A 93-year-old male weighing an estimated 55 kg (predicted body weight, as immobility precluded measurement of actual weight) was scheduled for cemented left hemiarthroplasty (HA) for a transcervical neck of femur fracture. The injury followed a ground-level slip at approximately 2:00 AM; the patient was discovered on the floor by family members with no direct witnesses. No preceding loss of consciousness, palpitations, or prodromal symptoms suggested a syncopal mechanism; the fall was attributed to PD-related gait instability. PD diagnosis had been established one week prior to admission, though symptom chronicity suggested longstanding unrecognised disease. Levodopa/carbidopa 100 mg/25 mg three times daily had been initiated at diagnosis.

Other comorbidities included hypothyroidism (levothyroxine 25 µg once daily), hypertension (losartan 50 mg once daily), and dyslipidaemia (rosuvastatin 10 mg once daily). The patient had a 6 pack-year smoking history, with cessation seven days prior to admission. Exclusive fluency in Newari, a language spoken primarily by the Newar ethnic community of Nepal, with minimal Nepali comprehension created a significant communication barrier. Preoperative history taking and written informed consent were facilitated through family members and an identified Newari-speaking hospital attendant.

Pain was assessed using the Abbey Pain Scale, validated for nonverbal and cognitively impaired patients, [6] yielding a score of 10, indicating moderate acute-on-chronic pain. Preoperative vital signs: blood pressure 140/90 mmHg, heart rate 84 beats min⁻¹ (regular), SpO₂ 96% on room air, afebrile. Airway examination: multiple missing teeth (none loose), Mallampati grade III in the supine position. Spinal examination confirmed feasibility in the lateral decubitus position.

Cardiology evaluation demonstrated normal left ventricular systolic function with an ejection fraction of 65%, no regional wall motion abnormalities, and grade I diastolic dysfunction. Laboratory investigations showed haemoglobin 14.4 g/dL. Thyroid function tests revealed T3 2.1 nmol/L, T4 12.6 nmol/L, and TSH 4.3 mU/L, consistent with adequately controlled hypothyroidism on current replacement therapy. Renal function tests, liver function tests, and coagulation parameters (PT/INR) were within normal limits. Electrocardiography and chest radiography were normal. Pelvic radiography confirmed the fracture (Fig. 1). Given the patient’s Parkinson’s disease and the recognised risk of oropharyngeal dysphagia, a formal preoperative swallowing assessment by a speech and language therapist was indicated but was not performed because of local resource constraints.

**Fig. 1:**
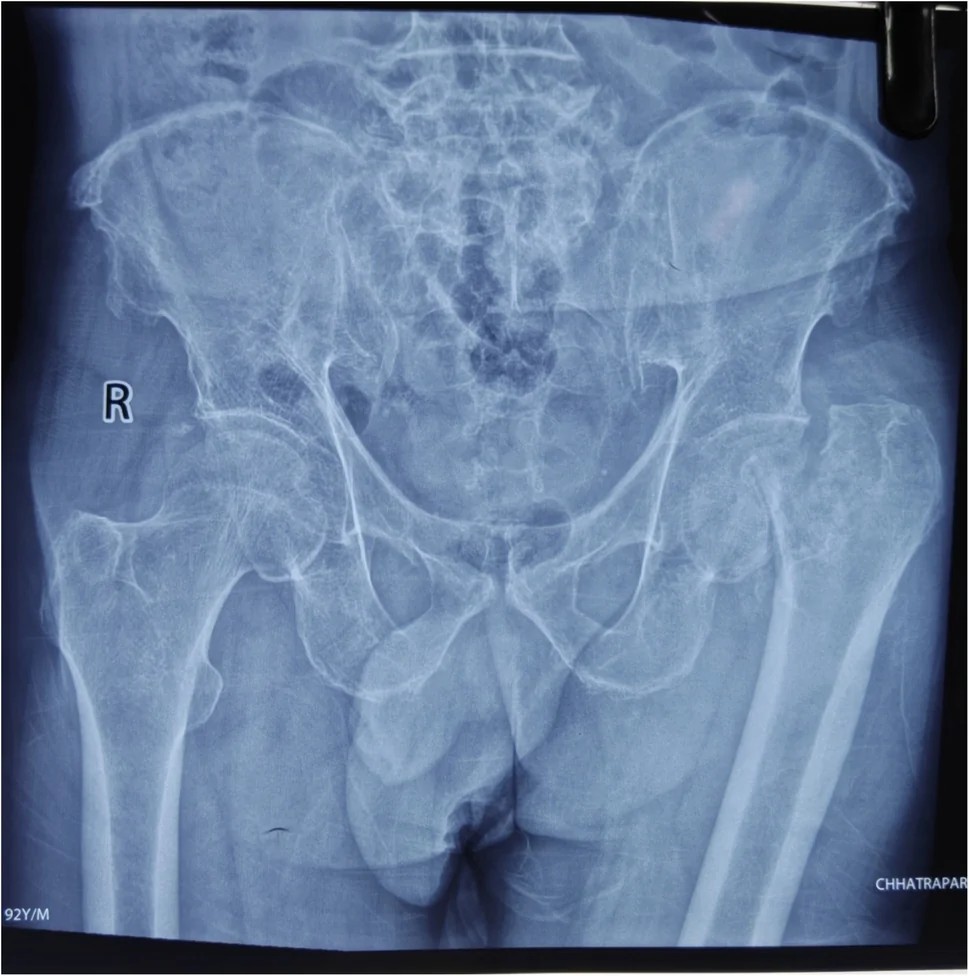
Preoperative anteroposterior pelvic radiograph demonstrating a left transcervical neck of femur fracture

The patient was classified ASA physical status III, reflecting substantive functional limitations with multiple comorbidities. Comprehensive family counselling addressed the risks of haemodynamic instability, respiratory compromise, postoperative delirium, postoperative neurocognitive dysfunction, and aspiration the latter of heightened concern given PD-associated possible oropharyngeal dysphagia. Nil per oral was maintained in accordance with ASA NPO guidelines. Routine medications were continued on the morning of surgery, with the exception of losartan, which was withheld to mitigate additive hypotensive risk. Levodopa/carbidopa was administered with a minimal sip of water at the scheduled morning dose to prevent dopaminergic interruption.

### Intraoperative management

Standard monitoring in accordance with ASA guidelines was established: continuous electrocardiography, non-invasive blood pressure monitoring (5-minute intervals), pulse oximetry and temperature targeting 35–36 °C. An 18-gauge intravenous cannula was placed. Warming blankets and a forced-air warmer were applied to prevent hypothermia.

Spinal anaesthesia was performed in the supported sitting position at the L3–L4 interspace using a 25-gauge Whitacre pencil-point needle. The sitting position was chosen to facilitate landmark identification, given the elevated risk of inadvertent movement in the lateral position due to pain and PD-related rigidity. A total of 3 mL of 0.5% hyperbaric bupivacaine (15 mg) combined with dexmedetomidine 5 µg was administered intrathecally. A bupivacaine dose of 15 mg reflects the conventional adult dose requested by the orthopaedic team for anticipated longer duration in surgery; however, in retrospect, a reduced dose of 4–8 mg with intrathecal adjuncts would have been more appropriate in this nonagenarian to limit the extent of sympathetic block, this report illustrates an important learning point discussed below. Co-loading with 500 mL Ringer’s lactate was administered concurrently.

Sensory blockade was assessed using bilateral pinprick testing (22G needle) at 2-minute intervals along the midclavicular line. A peak sensory block level of T6 was achieved before surgical incision, confirmed by loss of pinprick sensation and patient verbal response with assistance from a Newari-speaking anaesthesia assistant. The cemented HA lasted approximately 90 min, which was shorter than expected.

Total intraoperative fluid administration was 2000 mL. Estimated blood loss was 300 mL. Urine output was 150 mL over 90 min (approximately 1.8 mL kg − 1 h − 1 based on predicted body weight), indicating adequate renal perfusion. Intraoperative haemodynamic remained within clinically acceptable limits: blood pressure ranged from 85–130/55–90 mmHg. Three episodes of intraoperative hypotension (systolic blood pressure < 90 mmHg) were each managed with intravenous mephentermine 6 mg (total 18 mg) alongside 100–200 mL crystalloid boluses. Heart rate ranged from 62 to 80 beats min⁻¹, SpO₂ remained 98–100% with supplemental oxygen via nasal prongs (2 L min⁻¹), and temperature was maintained between 35.0 and 36.5 °C with active warming throughout.

As this was the last scheduled case of the day, the patient had been fasting for more than eight hours. Therefore, intraoperative blood glucose, checked at approximately 60 min postinduction, was 55 mg/dL, meeting the threshold for hypoglycaemia (blood glucose < 70 mg/dL). Forty millilitres of 50% dextrose was administered intravenously, with a subsequent check confirming normalisation. No further hypoglycaemic episodes occurred.

### Postoperative management

Motor and sensory recovery from spinal anaesthesia was complete at approximately four hours postoperatively, which was also assisted via the Newari-speaking attendant. Monitoring in the post anaesthesia care unit comprised continuous ECG, blood pressure, SpO₂, and hourly blood glucose for the first four hours. For postoperative analgesia, the patient received a single dose of tramadol 50 mg intravenously alongside ondansetron 4 mg for antiemetic prophylaxis; no benzodiazepines were administered at any point during the hospitalisation.

Because the patient was kept NPO, his levodopa/carbidopa was temporarily withheld immediately postoperatively. Delirium screening was performed every 12 h using the Confusion Assessment Method (CAM) throughout the postoperative period. By the first postoperative day, he grew visibly apathetic, slow, and poorly responsive. A non-contrast computed tomography scan of the head excluded acute intracranial pathology (Fig. 2), with findings consistent only with age-related changes. After exclusion of structural neurological causes, the patient’s acute, fluctuating decline in attention and consciousness met the CAM screening criteria for hypoactive delirium. [7] Once his levodopa/carbidopa was resumed via the newly placed nasogastric tube, the patient’s apathy and psychomotor slowing steadily improved. This clear clinical reversal strongly implicates acute dopaminergic interruption as the primary driver of his delirium. We managed the patient’s delirium by prioritising essential non-pharmacological interventions, such as family presence, reorientation, and mobilisation. While AGS/ACS guidelines strongly advocate for structured, multicomponent delirium prevention protocols, full perioperative implementation was regrettably precluded by local resource constraints. [8].

**Fig. 2:**
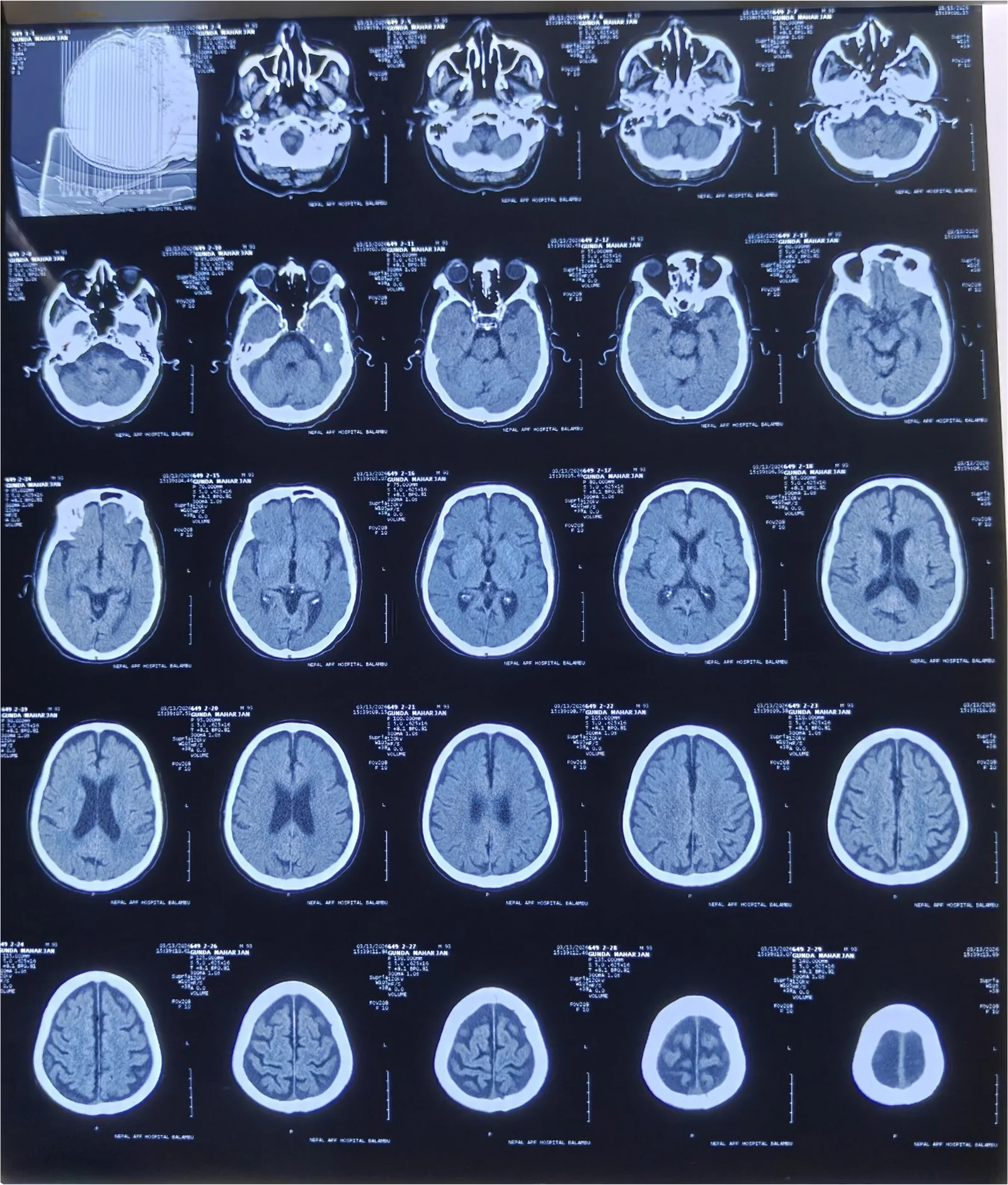
Noncontrast CT scan of the head demonstrating age-related cerebral changes (generalised cerebral atrophy and periventricular white matter changes) with no evidence of acute intracranial pathology

On postoperative day three, the patient developed clinical and radiological signs of aspiration pneumonia. Notably, this was not a result of opioid-induced vomiting or silent aspiration from his Parkinson’s disease. Instead, the complication occurred when well-intentioned family members fed him orally during a brief nursing absence, despite the presence of a functioning nasogastric tube. Broad-spectrum antibiotic therapy (piperacillin–tazobactam) and chest physiotherapy were initiated. It must be acknowledged that no formal preoperative swallowing assessment was performed, a limitation that is further discussed below. Antiparkinsonian medications were continued without interruption throughout. The patient was discharged on postoperative day 10 in a stable condition with outpatient follow-up arranged (Table 1).

**Table 1:** Perioperative management summary for PD patients undergoing hip fracture surgery

Domain	Evidence-Based Recommendation	Practice in This Case / Learning Point
Intrathecal bupivacaine dose (nonagenarian)	4–8 mg with intrathecal adjuvant (e.g. fentanyl 20 µg or dexmedetomidine 5 µg); ED95 as low as 1.7 mg reported with fentanyl. [11, 13]	15 mg used (conventional adult dose); resulted in T6 block and 3 hypotensive episodes. Dose reduction recommended in future
Perioperative dopaminergic continuity	Continue levodopa/carbidopa perioperatively; plan nasogastric route preoperatively; avoid any interruption [3, 4]	Morning dose given; postoperative delay pending NG insertion precipitated hypoactive delirium, resolved with enteral resumption
Multicomponent delirium prevention	Early mobilisation, cognitive orientation, sensory optimisation, sleep hygiene, nonopioid analgesia, family presence; reduces risk by 47–53%. [7, 8]	Measures implemented reactively; prospective structured protocol not in place preoperatively (acknowledged limitation)
Preoperative swallowing assessment	Formal SLT assessment mandatory in all PD patients; dysphagia in 40–80%; dietary modification and caregiver education before surgery. [3, 4]	Not performed preoperatively; aspiration pneumonia occurred POD3 due to unsupervised family oral feeding (acknowledged limitation)
Vasopressor selection for postspinal hypotension	Phenylephrine or norepinephrine preferred (titratable, predictable) for postspinal hypotension. [17]	Mephentermine 6 mg × 3 used (resource-limited setting); effective but suboptimal per current evidence

*Abbreviations*: *NG* Nasogastric, *PD* Parkinson’s disease, *POD* Postoperative day, *SLT* Speech and language therapy

## Discussion

This case emphasises conduct of spinal anaesthesia in a 93-year-old patient with PD undergoing hemiarthroplasty for a neck of femur fracture with essential learning points. The patient’s age was substantially greater than the reported mean of 73.2 ± 9.1 years for hemiarthroplasty recipients in Nepal, [9] and his combined burden of PD, communication barriers, intraoperative hypoglycaemia, postoperative hypoactive delirium and postoperative aspiration pneumonia renders this case educationally valuable across several domains.

Regional anaesthesia confers important advantages over general anaesthesia in frail elderly patients: avoidance of airway manipulation, reduction of respiratory complications, lower rates of PONV, preservation of gastrointestinal motility via sympathetic blockade, and reduced opioid requirements. [5] In patients with Parkinson’s disease, minimising opioid exposure and postoperative nausea and vomiting helps maintain uninterrupted oral levodopa/carbidopa therapy, as no parenteral formulation is available.

### Learning point: bupivacaine dosing in nonagenarians

The bupivacaine dose used in this case (15 mg) reflects conventional dosing, [10] chosen in anticipation of a potentially prolonged surgical duration, as discussed with the orthopaedic team; however, the procedure was completed earlier than expected. Current evidence indicates that lower doses are more appropriate in nonagenarians due to reduced CSF volume and increased sensitivity to neuraxial blockade, resulting in exaggerated spread and hypotension, as reflected by the T6 sensory level and intraoperative hypotension in this case. [11].

Hitka et al. reported that with intrathecal fentanyl (20 µg), the ED95 of bupivacaine in elderly patients undergoing hemiarthroplasty was only 1.7 mg, with a mean total dose of 4.35 mg, supporting ultra-low-dose spinal anaesthesia. [11] Similarly, Minville et al. demonstrated a 68% incidence of hypotension even with 7.5 mg bupivacaine in elderly patients. [12].

A reduced dose range of 4–8 mg bupivacaine with intrathecal adjuvants would have been more appropriate. Intrathecal fentanyl 20 µg is a well-established adjuvant that enhances analgesia without increasing sympathetic block and is a commonly used alternative to dexmedetomidine. [13].

### Learning point: dexmedetomidine risk–benefit considerations in Parkinson’s disease

While intrathecal dexmedetomidine (e.g., 5 µg) improves surgical conditions and prolongs sensory blockade, its administration remains off-label and carries distinct cardiovascular liabilities. A meta-analysis by Liu et al. reported a relative risk of 1.59 for bradycardia and 1.40 for hypotension, underscoring the absolute necessity for vigilant haemodynamic monitoring when this agent is selected. [14] In this patient, dexmedetomidine was deliberately chosen over fentanyl to avoid opioid-induced postoperative nausea and vomiting (PONV). Because levodopa/carbidopa lacks a parenteral formulation, any PONV-mediated interruption in oral intake directly threatens dopaminergic continuity and risks precipitating acute motor deterioration. This clinical trade-off accepting higher haemodynamic risks to safeguard essential oral therapy must be carefully weighed when selecting neuraxial adjuvants for patients with Parkinson’s disease.

Although this patient had no hepatic impairment, amide local anaesthetics such as bupivacaine undergo hepatic metabolism, and dose adjustment would be warranted in significant hepatic dysfunction.

While mephentermine was utilised due to resource limitations in this setting, current evidence strongly favours alpha-1 adrenergic agonists like norepinephrine or phenylephrine due to their predictable, titratable haemodynamic profiles and lower risk of tachyphylaxis. [17] Overall, this observation underscores that while standard doses may be feasible with vasopressor support, dose reduction combined with intrathecal adjuvants represents a safer and more predictable strategy for spinal anaesthesia in nonagenarian patients.

### Learning point: perioperative dopaminergic drug delivery

This case provides a clear clinical demonstration of the consequences of perioperative dopaminergic interruption. Antiparkinsonian medications should therefore be continued throughout the intraoperative and postoperative periods with minimal interruption to avoid motor deterioration and related complications. [3] Levodopa/carbidopa has no intravenous formulation; enteral delivery is the only available route. Any barrier to oral absorption-PONV, dysphagia, NPO status, impaired consciousness; therefore, it directly threatens therapeutic continuity. In this patient, postoperative withholding of levodopa (necessitated by NPO status and delayed nasogastric tube insertion) was followed by hypoactive delirium, which resolved upon enteral drug resumption. This temporal relationship strongly implicates dopaminergic interruption as the primary aetiology, distinct from purely multifactorial postoperative delirium. [3] A preoperative plan for alternative drug delivery routes (nasogastric or jejunal administration) must be established for every PD patient undergoing surgery. General anaesthesia was deliberately avoided: volatile agents and opioids impair dopaminergic transmission and complicate postoperative neurological assessment. [3].

### Learning point: recognition and prevention of hypoactive delirium

Hypoactive delirium is the most commonly missed subtype, presenting with psychomotor slowing, reduced responsiveness, and apathy, and is often mistaken for postanaesthetic fatigue or “quiet recovery. Systematic CAM (confusion assessment method) screening is essential in all elderly postoperative patients, as delayed recognition allows clinical deterioration. [7] In PD, this risk is compounded by concurrent dopaminergic withdrawal. Dopamine-blocking agents (haloperidol, metoclopramide, prochlorperazine) are contraindicated, as they can precipitate acute motor and autonomic crisis. Ondansetron, a selective 5-HT3 antagonist without dopamine receptor activity, is the antiemetic agent of choice in PD. Multicomponent non-pharmacologic interventions reduce delirium risk by 47–53%.^7^ The AGS/ACS guidelines strongly recommend these multicomponent interventions, which include early mobilisation, cognitive orientation, sensory optimisation, sleep enhancement, and pain management with nonopioids. The implementation of such structured protocols is essential to reduce delirium incidence in this highly vulnerable population, as prevention is consistently more effective than treatment. [8].

### Learning point: intraoperative hypoglycaemia in nondiabetic elderly patients

This patient was nondiabetic but vulnerable as he was scheduled for last case of the day, which resulted in a fasting period exceeding eight hours. Advanced age can reduce hepatic glycogen reserves and impair gluconeogenesis. Parkinson’s disease–related autonomic dysfunction may further blunt counter-regulatory responses to glucose metabolism and lead to neurocognitive dysfunction. [15] Blood glucose was checked ~ 60 min after spinal anaesthesia and hypoglycaemia was corrected promptly. Although not routine in nondiabetic patients, intraoperative monitoring was performed due to prolonged fasting, advanced age, and Parkinson’s disease. In elderly nondiabetic patients with prolonged fasting or autonomic dysfunction, glucose monitoring should be considered beyond diabetics, and preoperative carbohydrate loading (ERAS) may help reduce metabolic risk. [16].

### Learning point: aspiration pneumonia, contextualisation and practice implications

Oropharyngeal dysphagia occurs in 40–80% of patients with PD due to bradykinesia and rigidity of the pharyngeal musculature, and aspiration pneumonia is the leading cause of disease-related mortality in this population. [3, 4] Perioperative risk is further increased by postoperative depressed level of consciousness, opioid-induced nausea, vomiting, and respiratory depression, and prolonged immobilisation. In this instance, the aspiration was triggered by a well-meaning behavioural decision rather than a physiological complication. During a brief nursing absence, family members fed the patient orally despite a functioning nasogastric tube. This preventable event highlights a gap in postoperative care: inadequate caregiver education. Families of dysphagic patients should be clearly instructed to avoid unsupervised oral feeding. Dysphagia screening, speech and language therapy (SLT) consultation, and dietary modification should be initiated during preoperative assessment rather than after complications occur. [3] In retrospect, the absence of a formal preoperative SLT assessment represented a critical patient safety gap in this case. This complication underscores the importance of routine preoperative dysphagia screening, caregiver counselling, and supervised oral feeding in patients with Parkinson’s disease.

The patient’s exclusive fluency in Newari posed a significant safety challenge. While professional interpreters remain the international gold standard, utilising a Newari speaking hospital staff member for consent and perioperative communication provided a practical and effective safeguard within our resource constraints. [18] This case points to the lack of a formal communication protocol for minority-language patients. Future practice should include early interpreter identification and structured communication support rather than relying solely on ad hoc staff assistance. The Abbey Pain Scale was the appropriate validated tool for pain assessment in this nonverbal, cognitively impaired patient. [6].

### Limitations

Several limitations merit acknowledgement. First, the bupivacaine dose (15 mg) exceeded the evidence-based optimum for a nonagenarian, contributing to high sensory block and haemodynamic instability; doses of 4–8 mg with adjuvant supplementation represents best practice in this age group. Second, intrathecal dexmedetomidine was used as an adjuvant; while supported by published evidence, its use in this context remains off-label, and its contribution to haemodynamic instability cannot be entirely excluded. Third, non-pharmacological delirium prevention measures were applied reactively rather than through a prospective, risk-stratified protocol — a missed opportunity to reduce delirium incidence. Fourth, no formal preoperative swallowing assessment by a speech and language therapist was conducted, despite the high prevalence of oropharyngeal dysphagia in Parkinson’s disease; this omission preceded the aspiration pneumonia event and constitutes a preventable patient safety gap. Given the well-established prevalence of dysphagia in Parkinson's disease, formal preoperative SLT assessment should have been performed and may have identified swallowing impairment before postoperative feeding was attempted. Fifth, the inherent limitations of a single case report preclude generalisation of findings; the observations and learning points presented here are hypothesis-generating and should be interpreted in the context of the broader published literature. Sixth, resource constraints in this setting precluded professional interpreter services, formal delirium prevention protocols, and comprehensive preoperative swallowing assessment, reflecting the realities of care delivery in resource-limited environments.

## Conclusion

Spinal anaesthesia can be employed in nonagenarians with Parkinson's disease and multiple comorbidities undergoing hip fracture surgery; however, this case underscores the critical importance of evidence-based dosing over mere feasibility. A reduced bupivacaine dose supplemented by intrathecal adjuncts should be the default approach to minimise sympathetic block extent. Perioperative dopaminergic interruption—even brief—can precipitate hypoactive delirium that resolves with early enteral levodopa resumption. Crucially, this case demonstrates that for both delirium and aspiration pneumonia, prevention must be prioritised over treatment. Achieving optimal outcomes requires a multicomponent, multidisciplinary approach that integrates structured family education, preoperative swallowing assessment by Speech and Language Therapists (SLT), proactive enteral medication planning, and established delirium prevention protocols.

## Supplementary Information

Supplementary Material 1.

Supplementary Material 2.

## Data Availability

All relevant data supporting the findings of this case report are included within the article.

## Abbreviations

ASA: American Society of Anaesthesiologists
CAM: Confusion Assessment Method
CT: Computed Tomography
ECG: Electrocardiography
ERAS: Enhanced Recovery After Surgery
HA: Hemiarthroplasty
NG: Nasogastric
NPO: Nil Per Oral
PD: Parkinson’s Disease
POD: Postoperative Day
PONV: Postoperative Nausea and Vomiting
PT: Prothrombin Time
SLT: Speech and language Therapy
SpO₂: Peripheral Capillary Oxygen Saturation
T3: Triiodothyronine
T4: Thyroxine (Tetraiodothyronine)
TSH: Thyroid-Stimulating Hormone
